# Paediatric single mitochondrial DNA deletion disorders: an overlapping spectrum of disease

**DOI:** 10.1007/s10545-014-9778-4

**Published:** 2014-10-29

**Authors:** Alexander Broomfield, Mary G. Sweeney, Cathy E. Woodward, Carl Fratter, Andrew M. Morris, James V. Leonard, Lara Abulhoul, Stephanie Grunewald, Peter T. Clayton, Michael G. Hanna, Joanna Poulton, Shamima Rahman

**Affiliations:** 1 Genetic Medicine, Central Manchester University Hospitals NHS Foundation trust, St Mary’s Hospital, 6th Floor, Oxford Road, Manchester, M 13 9WL UK; 2 Neurogenetics Unit, National Hospital for Neurology & Neurosurgery, Queen Square, London, WC1N 3BG UK; 3 Oxford Medical Genetics Laboratories, Oxford University Hospitals NHS Trust, The Churchill Hospital, Oxford, OX3 7LE UK; 4 40A Bagley Wood Road, Kennington, Oxford, OX1 5LY UK; 5 Metabolic Unit, Great Ormond Street Hospital NHS Foundation Trust, Institute of Child Health, Great Ormond Street, London, WC1N 3JH UK; 6 Genetics and Genomic Medicine, UCL Institute of Child Health, 30 Guilford Street, London, WC1N 1EH UK; 7 MRC Centre for Neuromuscular Diseases, UCL Institute of Neurology and National Hospital for Neurology and Neurosurgery, Queen Square, London, WC1N 3BG UK; 8 NDOG, Level 3, Women’s Centre, John Radcliffe Hospital, Oxford, Oxfordshire OX3 9DU UK; 9 Mitochondrial Research Group, Genetics and Genomic Medicine, UCL Institute of Child Health, 30 Guilford Street, London, WC1N 1EH UK

## Abstract

**Background:**

Single large-scale mitochondrial DNA (mtDNA) deletions (SLSMDs) are amongst the most frequently diagnosed mtDNA disorders in childhood, yet their natural history remains poorly understood. We report the natural history of a large multicentre cohort of such children.

**Methods:**

We reviewed case notes from three different UK centres to determine the clinical course of 34 patients (16 female, 18 male) with childhood-onset mitochondrial disease caused by SLSMDs. Kaplan–Meier analysis was used to compare survival of patients presenting with haematological features (Pearson syndrome) and those with nonhaematological presentations.

**Results:**

The most frequent initial presentation was with isolated ptosis (16/34, 47 %). Eleven (32 %) patients presented with transfusion-dependent anaemia soon after birth and were diagnosed with Pearson syndrome, whilst ten were classified as having Kearns–Sayre syndrome, three as having progressive external ophthalmoplegia (PEO) and seven as having PEO-plus. Three patients did not conform to any specific mitochondrial syndrome. The most frequently affected organ during the disease course was the kidney, with documented tubular or glomerular dysfunction in 17 of 20 (85 %) cases who had detailed investigations. SLSMDs were present in blood and/or urine cells in all cases tested, indicating that muscle biopsy is not necessary for diagnosis in the paediatric age range. Kaplan–Meier survival analysis revealed significantly worse mortality in patients with Pearson syndrome compared with the rest of the cohort.

**Conclusions:**

Mitochondrial disease caused by SLSMDs is clinically heterogeneous, and not all cases conform to a classical mitochondrial syndrome. Multisystem disease is the norm, with anaemia, renal impairment and endocrine disturbance being the most frequent extraneurological features. SLSMDs should be considered in the differential diagnosis of all children presenting with ptosis.

**Electronic supplementary material:**

The online version of this article (doi:10.1007/s10545-014-9778-4) contains supplementary material, which is available to authorized users.

## Background

The overall incidence of mitochondrial disease is uncertain but has been estimated to be as frequent as 1 in 5,000 births (Skladal et al. 2003; Schaefer et al. 2004), with single, large-scale mitochondrial DNA (mtDNA) deletions (SLSMDs) contributing to 10 % of primary mtDNA disorders (Lamont et al. 1998). These deletions tend to occur spontaneously, possibly arising from defective mtDNA repair mechanisms (Krishnan et al. 2008) or defective mtDNA replication (Shoffner et al. 1989). In some cases, rearrangements are complex and many include duplicated mtDNA species (Poulton et al. 1993; Poulton et al. 1989).

The cornerstone of diagnosis has traditionally been the recognition of one of the classical phenotypes: Pearson marrow–pancreas syndrome, Kearns–Sayre syndrome (KSS), chronic progressive external ophthalmoplegia (CPEO or PEO) or (C)PEO-plus. Pearson syndrome was originally defined as a sideroblastic anaemia with associated exocrine pancreatic dysfunction (Pearson et al. 1979) but is now recognised as a multisystem mitochondrial disorder in which the chief feature is sideroblastic anaemia (Manea et al. 2009). KSS is defined as PEO with pigmentary retinopathy, presenting before the age of 20 years, with at least one of these additional findings: high cerebrospinal fluid (CSF) protein content, cardiac conduction block or ataxia (Rowland 1983). Isolated PEO is considered a more benign single-system disorder affecting skeletal muscle and is characterised by ptosis, ophthalmoplegia and variably severe proximal limb weakness (Biousse and Newman 2001). The term CPEO-plus was coined by Drachman in 1968 (Drachman 1968) and is now used to describe patients with PEO who, whilst not fulfilling the criteria for KSS, have multisystem involvement (Di Mauro and Hirano 1993). While these clinical phenotypes have been the mainstay of recognition of SLSMD disorders, increasing experience has begun to demonstrate considerable overlap between these syndromes (Pitceathly et al. 2012, Manea et al. 2009; Yamashita et al. 2008; Grady et al. 2014).

We report a large multicentre paediatric-onset cohort that demonstrates the clinical overlap of SLSMD disorders presenting in childhood and adds weight to the view that the apparently distinct Pearson and Kearns–Sayre syndromes form part of a continuous spectrum of disease (Dimauro 2007; Lopez-Gallardo et al. 2009). Moreover, the findings reported in our study emphasise the importance of investigating apparently isolated ptosis presenting in childhood and of a detailed renal evaluation in all patients with SLSMDs.

## Patients and methods

We conducted a multicenter case-notes review of childhood-onset SLSMDs diagnosed between 1988 and 2011. All patients diagnosed with symptom onset before age 16 years were identified through the National Health Service (NHS) Highly Specialised Services-funded mitochondrial molecular diagnostic laboratories at the National Hospital for Neurology, London, and the Oxford Radcliffe Hospitals, Oxford. Molecular diagnosis of SLSMD was achieved in all cases by Southern blot and/or long-range polymerase chain reaction (PCR) of DNA extracted from peripheral blood leukocytes, urinary epithelial cells or skeletal muscle. All genetic studies were performed after informed consent from parents/legal guardians of the patients. Identification of the deletion break point was performed by Sanger sequencing with the use of appropriate primers where samples were available. Quantitation of mitochondrial deletion mutation load compared with the wild type was determined visually by experienced scientists. This visual method of quantitation on autoradiographs has been validated over a number of years by the blinded visual comparison of deletion load with that found using densitometry (Holt et al. 1989; McShane et al. 1991), and we previously established that this is associated with an operator error of 5 % compared with densitometry reading (unpublished observations).

AB or SR reviewed all case notes, and clinical data (including symptom onset, multisystem disease features, neuroimaging, muscle histology and histochemistry, blood and CSF biochemistry, respiratory-chain enzyme activities in muscle, genetic data) were collated using a structured pro forma. Statistical analysis was performed using the SPSS 16 (SPSS for Windows, Version 16.0. Chicago, IL, USA). The Bonferroni correction was used for multivariate regression analysis, and Kaplan–Meier analysis was performed for comparison of survival outcomes. Statistical significance was set at *p* <0.05. Ethical approval for the study was obtained from the National Research Ethics Committee London, Bloomsbury, UK.

## Results

### Demographics

Thirty-four patients were identified: 16 female and 18 male. There was no family history in any case except one set of identical twins, patients P and Q, who presented very similar clinical features (Table 1). Eleven patients were diagnosed with Pearson syndrome, ten with KSS, three with PEO and seven with PEO-plus. However, three patients (K, L and R) did not correspond to any of these classical phenotypes (Table 2). The details of their initial presentation are shown in Table 1.

**Table 1:** Demographics and clinical features at presentation

Patient	Sex	Onset	Age at diagnosis	Age at death	Age at last review (years)	Birth weight (kg)	Presenting complaint
A	F	Birth	1.5 months	5 years 3 months		2.5	Anaemia at birth
B	M	Birth	2 years 2 months	7 years 5 months		3.46	Poor feeding, then anaemia
C	M	Birth	7 years 10 months		8.5	3.4	Anaemia at birth
D	F	Birth	5 months	2 years 6 months		1.6 (Twin 2, 34 weeks’ gestation)	Anaemia at birth
E	F	Birth	1 months	4 months		1.54	IUGR/pancytopenia/diabetes mellitus/RVH
F	M	Birth	4 years 8 months		6	3.35	Ptosis + anaemia
G	F	Birth	2 months	14 months		1.7 (Twin 1, 34 weeks’ gestation)	Anaemia
H	M	2 months	13 months	2 years 4 months		3.7	Intermittent dyspnoea + anaemia
I	F	5 months	2 years 8 months	4 years 5 months		3	Anaemia
J	M	16 months	2 years		6	NA	Anaemia
K	F	6 months	21 months	16 years 2 months		3.9	Failure to thrive + diarrhoea
L	M	2 years	14 years		24	NA	Fanconi syndrome + rickets
M	M	2 years	6 years	11 years 5 m		2.9	Left-sided ptosis
N	F	2 years 6 months	6 years		10	NA	Ptosis
O	M	3 years	6 years	21 years		3.8	Low appetite/low energy/hyponatraemia
P	M	4 years	8 years		12	1.7 (Twin 2)	Ptosis at 4 years, adrenal insufficiency at 5 years
Q	M	5 years	8 years		12	2.8 (Twin 1)	Adrenal insufficiency investigated after twin 2’s diagnosis
R	M	Birth	3 months	1 year 9 months		2.32	Poor feeding, hypoglycaemia, faltering growth, lactic acidosis
S	F	12 years	17 years		25	3.05	Ptosis onset at 12 years
T	F	8 years	12 years		12	NA	Tremor + migraine at 8 years, ptosis at 12 years
U	F	5 years	19 years		19	NA	Ptosis
V	F	7 years	8 years		20	NA	Ptosis
W	M	5 years	10 years		15	NA	Recurrent inflammation of the eye
X	F	15 years	25 years		25	NA	Ptosis from early childhood
Y	M	14 years	24 years		24	NA	Ptosis
Z	M	8 years	13 years		13	2.2	Ptosis
AA	M	11 years	13 years		13	3.06	Ptosis
AB	F	5 years	9 years		15	NA	Ptosis
AC	M	6 years	13 years		17	3.85	Ptosis
AD	F	7 years	12 years		22	NA	Ptosis
AE	F	7 years	18 years		25	NA	Ptosis, then muscular weakness at 14 years
AF	M	5 years	8 years		13	2.5	Short stature, poor appetite
AG	M	4 months	5 months		2.5	3.96	Anaemia and failure to thrive
AH	F	9 years 6 months	11 years		15	2.28	Ptosis

*IUGR* intrauterine growth restriction, *RVH* right ventricular hypertrophy, *NA* not available

**Table 2:** Genotypic data

Patient	Clinical diagnosis	Tissue investigated	Deletion breakage points (bp)	Size (kb)	Number of tRNA genes deleted	% heteroplasmy
A	Pearson	Bone marrow	Common del (8473→13447)	4.97	5	80
B	Pearson	Muscle	12102→14113	2.01	3	85
C	Pearson	Muscle	Common del (8473→13447)	4.97	5	90
D	Pearson	Blood	NA	5^a^	NA	NA
E	Pearson	Muscle	Common del (8473→13477)	4.97	5	90
F	Pearson	Blood	7983→13983	6	6	86
G	Pearson	Blood	Common del (8474→13447)	4.97	5	70
H	Pearson	Blood	NA	2.7^a^	NA	70
I	Pearson	Blood	Common del (8473→13446)	4.97	5	70
J	Pearson	Blood	NA	>5^a^	NA	50
K	Unclassified	Blood	NA	5.5^a^	NA	50
L	Unclassified	Blood	NA	4.2^a^	NA	NA
M	KSS	Blood	NA	7^a^	NA	NA
N	KSS	Blood Urine	7909→13378	5.46	6	70 85
O	KSS	Blood	NA	3.9^a^	NA	
P	PEO+	Blood	7771→15406	7.6	7	60
Q	PEO+	Blood	7771→15406	7.6	7	60
R	Unclassified	Blood	Common del (8467→13447)	4.96	5	85
S	KSS	Muscle	Common del (8473→13447)	4.97	5	80
T	KSS	Blood	Common del (8482→13477)	4.97	5	NA
U	PEO	Muscle	Common del ((8473→13477)	4.97	5	45
V	KSS	Blood Muscle	Common del (8473→13477)	4.97	5	55 45
W	KSS	Blood	Common del (8473→13477)	4.97	5	50
X	PEO	Muscle	Common del (8473→13477)	4.97	5	60
Y	PEO+	Muscle	Common del (8483→13477)	4.97	5	60
Z	KSS	Urine	Common del (8474→13477)	4.97	5	50
AA	PEO+	Blood	NA	5^a^	NA	NA
AB	PEO+	Blood	NA	3.6^a^	NA	NA
AC	KSS	Blood	NA	7.5^a^	NA	45
AD	PEO	Muscle	NA	4.4^a^	NA	75
AE	PEO+	Muscle	Common del (8473→13477)	4.97	5	NA
AF	KSS	Muscle	6133→14092	7.96	11	55
AG	Pearson	Blood	Common del (8473→13477)	5	5	80
AH	PEO +	Blood	NA	5.1^a^	NA	25

*del* deletion, *KSS* Kearns–Sayre syndrome, *PEO* progressive external ophthalmoplegia, *PEO* + PEO with additional clinical features, *NA* not available

^a^Approximate size

### Initial presentation

**Fig. 1:**
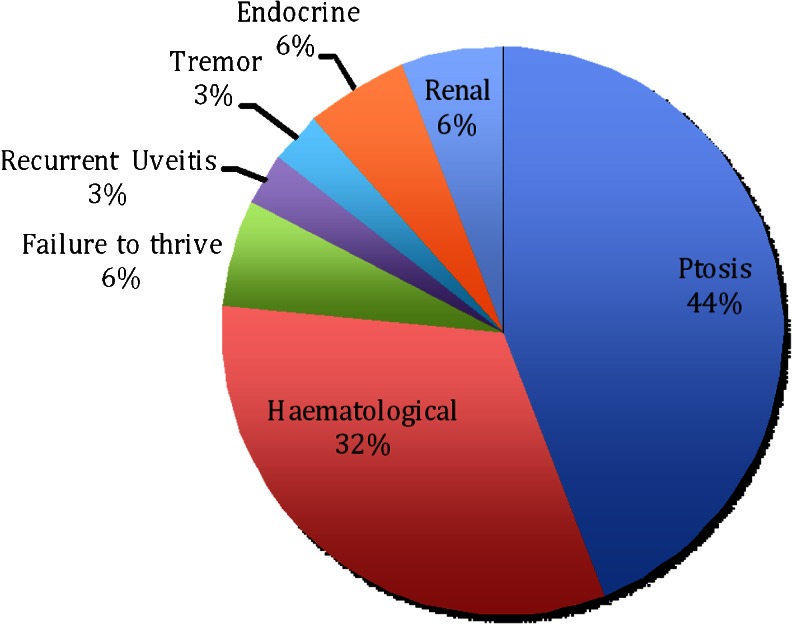
Clinical features at presentation. Initial clinical problems in 34 patients with childhood-onset mitochondrial disease caused by single large-scale mitochondrial DNA deletions

The initial symptoms at presentation are illustrated in Fig. 1. In 42 % of patients (16 of 34), the first symptom was ptosis, while haematological manifestations associated with Pearson syndrome were the second most frequent presenting feature, occurring in 32 % (11 of 34). Median age at presentation was 1 week [interquartile range (IQR) 3 months] for these 11 patients, and 6 years (IQR 5 years) for those not diagnosed with Pearson syndrome.

### Survival

**Fig. 2:**
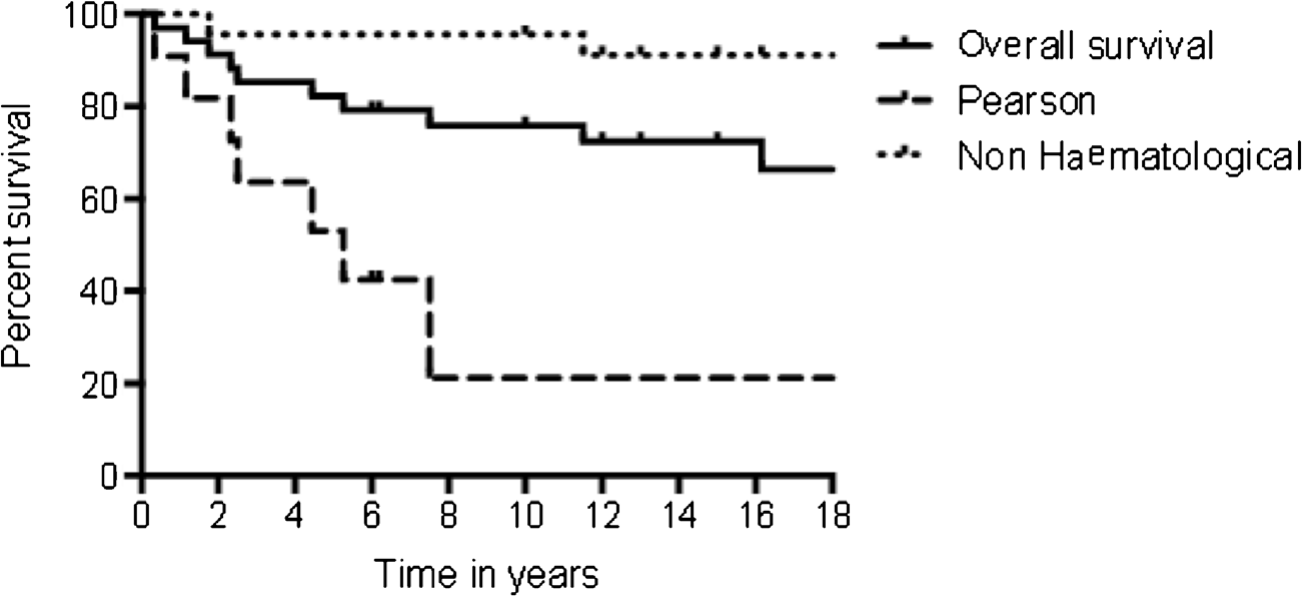
Kaplan–Meier survival graph showing overall survival to 18 years of 34 patients with single large-scale mitochondrial DNA deletions, compared with subgroups with a diagnosis of Pearson syndrome (*n* = 11) and those without haematological involvement. (*p* < 0.001 Mantel–Cox log-rank test.)

Overall, 11 patients died: seven with Pearson syndrome, two with KSS and two with atypical presentations. Kaplan–Meier survival analysis (Fig. 2) revealed a significant difference (*p* = 0.01) in mortality between those with Pearson syndrome when compared with the other clinical phenotypes. Survival at 18 years was 22 % for Pearson syndrome and 73 % for patients with other clinical presentations.

### Haematological involvement

Table S1 details the haematological manifestations observed in the 11 patients diagnosed with Pearson syndrome. Most of these cases presented soon after birth with transfusion-dependent anaemia. Neutropaenia developed in ten patients, and thrombocytopaenia was documented in eight cases. Bone marrow aspirates were performed in ten patients; all had vacuolization of haematopoietic precursors, although classical ringed sideroblasts were observed in only seven cases. No evidence of impaired haematopoeisis was documented in the other 23 patients.

### Gastrointestinal and endocrine involvement and growth

Birth weight was <3rd centile in six of 22 patients whose birth weights were available. It is to be noted that two of these six patients, D and G, were both born prematurely at 34 weeks; two patients, B and F, were second babies of twin pregnancies. The weight of 19 of 30 patients, for whom serial auxology was available, fell below the 3rd centile during follow-up (Table S2).

Four patients with Pearson syndrome had a history of recurrent diarrhoea, three of whom had reduced faecal elastase suggesting pancreatic insufficiency (Table S2). Overall, five of 16 patients tested had reduced faecal elastase, four of whom fulfilled criteria for classical Pearson syndrome. However, patient K never had any haematological problems (Morris et al. 1997). Patient K also developed a severe enteropathy with marked inflammatory cell infiltrate at age 4 years, necessitating parenteral nutrition for 6 months. Patient M developed severe abdominal pain, nausea, vomiting and diarrhoea at 10 years and had documented gastroparesis on electrogastrography and gastric-emptying scintigraphy studies. Of note, patient E, who had diabetes mellitus but no symptoms suggestive of malabsorption, had a hypoplastic pancreas on autopsy.

Endocrine dysfunction was a frequent occurrence: three patients (P, Q and W) had cortisol deficiency requiring steroid supplementation, one (A) had hypothyroidism, three (J, W, V) had hypoparathyroidism and nine had glucose tolerance tests indicative of diabetes mellitus. Of these nine, two patients (A and G) had concurrent pancreatic exocrine insufficiency suggesting global pancreatic dysfunction. Patients M, W and AF were shown to have growth hormone insufficiency and had a good clinical response to growth hormone supplementation.

### Renal disease

Impairment of renal function defined as either a reduction of glomerular filtration rate (GFR), measured by isotope excretion, or an abnormal elevation of urinary tubulopathy markers retinol binding protein (RBP) (Bernard et al. 1987) or N-acetyl-3-glucosaminidase (NAG) (Vaidya et al. 2008), i.e. an abnormal RBP/creatinine and/or an abnormal NAG/creatinine ratio, was observed in 14 of 20 patients in whom renal function had been investigated in detail (Table S3). Five of eight patients tested had abnormal GFRs, four of whom (A, K, L and W) also showed elevation of urinary tubulopathy markers suggesting global impairment of both filtration and tubular function. Patient K developed end-stage renal failure. Of the 16 patients who had NAG and RBP/creatinine ratios examined, both were elevated in 13, NAG in two and one was normal for both. In addition to the 20 patients who had formal investigations, two (G and H) were deemed likely to have had a tubulopathy given their combination of aminoaciduria and polyuria; however, this was never formally investigated. Four of seven renal biopsies were normal histologically despite severely reduced GFRs in three of these cases, while two biopsies revealed calcium deposition and the seventh showed cystic dilatation.

### Cardiac function

Cardiac function was assessed using electrocardiogram (ECG) (29 patients) and echocardiogram (28 patients), and abnormalities were detected in 13. Rhythm disturbances were found in nine of the 29 patients examined, including complete heart block in five (A, N, O, V and AF) at 5, 9, 12, 13 and 8 years, respectively. Patient A had a heart rate of only 20 beats per minute prior to pacing and died 3 weeks after developing complete heart block (Rahman et al. 2000). Patient N, alive 26 months after insertion of pacemaker, has severe cardiomyopathy with inter- and intraventricular dyssynchrony, dyskinetic ventricular septum and fractional shortening of 18 % despite high-dose lisinopril therapy. Two patients (I and W) developed incomplete right bundle branch block, a known precursor to more severe pathogenic conduction defects (Riera et al. 2008), having previously had normal ECGs. Other abnormalities noted included supraventricular disturbances (Y and L), first-degree block (Z), left ventricular hypertrophy (LVH) (K and R) and right ventricular hypertrophy (RVH) (H).

### Neurological features and neuroimaging

The most frequent neurological manifestation was ptosis, affecting 22 patients, although only nine had frank external ophthalmoplegia (Table 3). Interestingly, all of those who presented with ptosis, except patients W and Y, were given an initial diagnosis of congenital ptosis despite only patient F being symptomatic before the age of 1 year. Retinal dystrophy was observed in 13 patients, while patients B, L and W had corneal thickening, a previously unrecognised manifestation of SLSMD diseases. A delay in achievement of gross motor milestones was seen in nine of 34 patients, while 18 had clinical signs of neurological involvement, including eight with hypotonia, nine with reduced power and two with ataxia. Only three patients were reported to have seizures (Table 3).

**Table 3:** Neurological features, neuroimaging and muscle biopsy findings

Patient	Gross motor development	Signs of PEO or ptosis	Ophthalmology	Hearing	Seizures	Hypotonia/ movement disorder	MRI/CT^a^	Muscle histology and respiratory-chain enzymology
A	Delayed	Decreased right abducens at 5 years	Normal	NA	No	No	NA	NA
B	Normal	No	Corneal thickening	NA	No	No	NA	NA
C	Normal	No	Retinitis pigmentosa 6 years + decreased ERG	NA	NA	NA	CT normal 6 years	At 8 years: RRF, COX-neg fibres, glycogen filled vacuoles Normal RC enzymology
D	Normal	No	NA	NA	No	NA	NA	NA
E	Delayed	No	Normal	NA	Yes	Generalized from birth	NA	NA
F	Delayed	Ptosis from birth	Retinal dystrophy at 5 years	NA	NA	Generalized at 5 years	NA	Numerous RRF + COX-neg fibres Low complex IV activity
G	NA	No	NA	Normal	NA	NA	NA	NA
H	Normal	No	Normal	NA	NA	NA	NA	Normal
I	Normal	No	Normal	NA	No	No	NA	NA
J	Normal	No	NA	NA	NA	NA	Mild generalized white-matter disorder	NA
K	Delayed	No	Loss of vision at 7 years, retinal dystrophy	Cochlear implant at 7 years	No	Generalized	Normal at 2 years CT at 10 - subcortical white matter hypodensity In globus pallidus	Normal
L	No	No	Increased vascularity of corneas/corneal oedema 8 years	Bilateral high tone HL at 8 years	NA	NA	Normal at 14 years	Few RRF, scattered COX-neg fibres Low complex IV activity
M	Normal	Ptosis from 2 years + mild lateral rectus palsy 7 years	NA	Mild right high frequency HL	NA	NA	Bilateral calcification and T2 high signal in head of caudate	RRF, rare COX-neg fibres (4.5 %) Normal RC enzymology
N	Delayed	Ptosis from 3 years	NA	Bilateral high frequency HL	No	NA	Symmetrical signal abnormalities in cerebellar white matter, brainstem, globus pallidus and thalamus	NA
O	Delayed	Ptosis and lateral ophthalmopegia	Mild pigmentary changes but normal ERG	NA	NA	Generalized at 6 years	Normal	RRF + rare COX-neg fibres, excess lipid Enzymology not performed
P	Normal	Ptosis from 2 years	Mild Pigmentary changes	Normal	NA	NA	NA	NA
Q	Normal	No	NA	NA	NA	NA	Bilateral basal ganglia changes	NA
R	Delayed	No	Retinal dystrophy	Bilateral high tone HL	No	Generalized from birth	Polymicrogyria, hypoplasia of cerebellum	RRF + COX-neg fibres Decreased complex IV activity
S	Normal	Ptosis 12 years	Retinal dystrophy—pigmentary retinal changes seen at 16 years	Normal	No	Some ataxia, normal tone	Normal	RRF + COX-neg fibres Low complex I activity
T	Normal	Ptosis 12 years	Retinal dystrophy/ RP	NA	Yes	Generalized weakness and tremor at 12 years	Bilateral signal change thalami, pons, cerebellar peduncle, dentate nuclei, posterior medulla. Poor myelination	NA
U	Normal	Ptosis at 7 years	No	Normal	No	NA	NA	RRF + COX-neg fibres with increased lipid
V	Normal	Ptosis at 7 years, external ophthalmoplegia at 8 years	Pigmentary retinopathy 14 years	Bilateral high tone HL at 14 years	No	Ataxia from 12 years	NA	NA
W	Normal	Ptosis at 10 years, external ophthalmoplegia at 11 years	Corneal oedema at 5 years and recurrent inflammation	Bilateral HL at 7 years	No	Weakness and episodes of myopathy with raised CK	Bilateral signal changes affecting globus pallidi, midbrain, pons and cerebellar dentate nuclei. Some frontal white mater changes	NA
X	Normal	Ptosis at 15	No	NA	NA	Proximal Weakness from 24 years	NA	RRF + COX-neg fibres Normal RC enzymology
Y	Normal	Ptosis at 14	No	NA	NA	Proximal weakness from 25 years	NA	RRF + COX-neg fibres Low complex IV activity
Z	Normal	Ptosis at 8 years, Ophthalmoplegia 12 years	Yes	Bilateral high tone HL at 14 years	Yes at 4 years	No	Normal	NA
AA	Normal	Ptosis 11 years, Ophthalmoplegia 12 years	Normal	Normal	No	Distal weakness 12 years	NA	NA
AB	Mild delay	Ptosis	Pigmentary retinopathy 9 years	Bilateral high tone HL	NA	Hypotonia	NA	RRF + COX-neg fibres Low complex IV activity
AC	Normal	Ptosis 6 years, Ophthalmoplegia 13 years	Pigmentary retinopathy 13 years	NA	NA	NA	NA	NA
AD	Normal	Ptosis at 7 years	NA	NA	NA	Hypotonia age 12 years	NA	NA
AE	Normal	Ptosis at 7 years	Pigmentary retinopathy 18 years	NA	NA	Proximal myopathy at 14 years	NA	NA
AF	Delayed from infancy	Ptosis at 8 years + ophthalmoplegia	Normal	Bilateral high tone HL	NA	NA	Symmetrical abnormalities in globus pallidus, thalami and dorsal aspect of midbrain and pons	Some ragged blue fibres on combined COX/SDH stain, excess lipid, several necrotic fibres. Low complex I, III and IV activities
AG	Normal	NA	NA	NA	NA	NA	NA	NA
AH		Ptosis present at diagnosis	Pigmentary retinopathy				NA	

*CK* creatine kinase, *COX-neg* cytochrome oxidase negative, *CT* computed tomography, *ERG* electroretinogram, *HL* hearing loss, *MRI* magnetic resonance imaging, *RC* respiratory chain, *RRF* ragged red fibres, *SDH* succinate dehydrogenase, *NA* not available

^a^MRI changes documented unless otherwise stated

Ten of 15 patients tested had sensorineural hearing loss, including patient K who required cochlear implants (Table 3). Brain magnetic resonance imaging (MRI) was abnormal in nine (J, K,M, N, Q, R, T, W and AF) of 13 patients examined (Table 3). In eight cases, the predominant finding was of basal ganglia changes, but six also had white matter changes, whilst patient R had changes suggestive of a neuronal migration defect. Neuroimaging in the four patients (J, M, N and Z) with low levels of CSF 5-methyltetrahydrofolate (5-MTHF) revealed white matter lesions in the three severely affected, but patient Z, whose level was only just below the normal range, had a normal scan.

### Muscle histology and histochemistry

Muscle biopsies were performed in 14 patients and were abnormal in 12 cases (Table 3). Ragged red fibres were observed in all 12 abnormal biopsies, whilst cytochrome oxidase (COX)-negative fibres were seen in 11. Excessive lipid was observed in seven, while abnormalities in mitochondrial morphology on electron microscopy were seen in six. The two other histologically normal biopsies were both small samples and in the case of patient K was performed at the relatively early age of 9 months, while electron microscopy was not performed in patient H.

### Biochemistry

Blood lactate was raised (>2.0 mmol/l) in the majority (21 of 30, 70 %) of patients tested (Table S4). Of the 20 who had concurrent amino acid profiles performed, 15 had an accompanying rise in plasma alanine to 492–889 μmol/L (reference <450 μmol/l). Plasma alanine was elevated in 15 patients, but patients M, Q and W had no accompanying rise in lactate. Raised levels of lactate were noted in the urine of all patients except W. Patient M also had a raised plasma proline at 323 μmol/L (reference 85–290 μmol/l). Urinary organic acid analysis revealed an increase in 3-hydroxybutyrate in ten of 17 patients tested, suggestive of a shift in cellular redox potential to a more reduced state. Two patients (B and R) had raised levels of tricarboxylic acid cycle metabolites. CSF lactate was elevated (>1.8 mmol/L) in all ten patients tested. CSF protein was markedly elevated in all nine patients for whom data was available, in keeping with a diagnosis of KSS, although one of these (R) did not fulfil diagnostic criteria for KSS, since there was no ophthalmoplegia. All four patients who had CSF 5-MTHF determined had low levels, although two cases (J and Z) had initial normal values but later had undetectable levels (at the ages of 6 and 15 years, respectively).

### Genetics

Of the 34 patients in this cohort, the diagnosis of SLSMDs was established in 22 by analysis of blood DNA. Of the other 12, muscle was analysed in ten and urinary epithelial cells and tissue obtained from a bone marrow aspirate in one case each (Table 2). Breakage points were determined for 22 patients: 12 in blood, eight in muscle and one each in tissue from a bone marrow aspirate and urinary tract epithelial cells. Of these 22, 16 had the common 4.97 kb mtDNA deletion. There was no correlation between age of presentation and size of deletion in these 22 patients, but there was a statistically significant but weak correlation between per cent of deletion found in tissue and age at presentation (*p* = 0.04, *R* ^2^ = 0.45.) We observed no correlation between age at presentation and number of total RNA (tRNA) genes deleted or *MT-CYB* deletion in these 22 patients.

## Discussion

While Kearns and Sayre’s original clinical description was published in 1958 and Pearson’s was published in 1979 (Kearns and Sayre 1958; Pearson et al. 1979), knowledge that mtDNA deletions were responsible was only established in 1988 (Holt et al. 1988). Subsequently, many individual case reports and case series have been published that increasingly suggest a clinical overlap between these historically different phenotypes (Manea et al. 2009; Yamashita et al. 2008; Pitceathly et al. 2012; Grady et al. 2014; McShane et al. 1991).

All 34 patients described here had single mtDNA deletions: 11 (A–J and AG) presented with varying degrees of anaemia and haematological dysfunction and fulfilled diagnostic criteria for the Pearson marrow–pancreas syndrome. However, pancreatic exocrine dysfunction was only documented in four: A, B, G and I (Table S2). Even in those with pancreatic dysfunction, the dysfunction was first documented at least 2 years after the initial presentation with anaemia, providing further evidence that the presence of pancreatic exocrine dysfunction should not be considered as an essential diagnostic criterion for Pearson syndrome. Our findings are in agreement with previous observations that one third of Pearson syndrome patients present solely with anaemia (Manea et al. 2009), with pancreatic dysfunction at presentation in only 12.7 % of cases, although this increases to 18 % by the age of 4 years (Lee et al. 2007). Low birth weight has previously been reported to be the most common nonhaematological finding in Pearson syndrome, affecting up to 63 % of patients (Manea et al. 2009). While at first glance our cohort might appear to reinforce these observations, with four of ten being <3rd centile at birth, on closer examination, two of these patients (D and G) were born prematurely at 34 weeks’ gestation, while patients D and P were second twins.

**Fig. 3:**
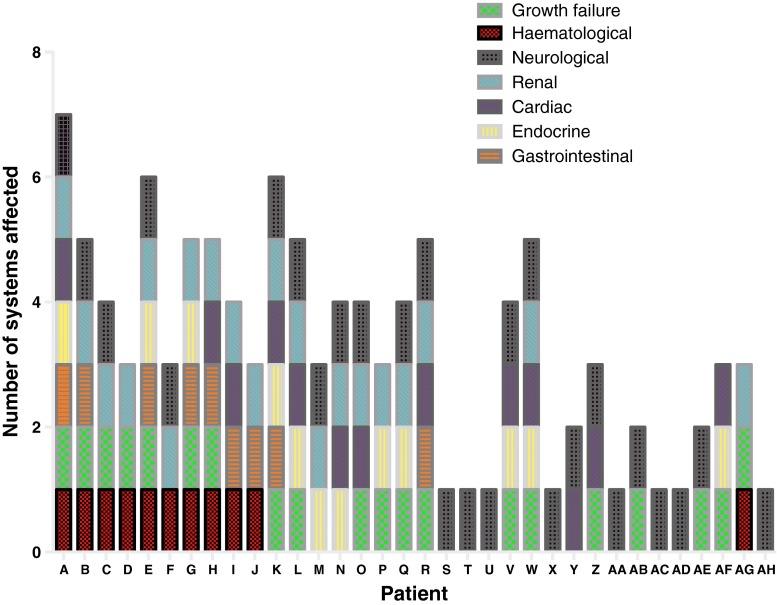
Number of different systems affected in each patient and impact on growth

A novel finding of our study is the extent of multisystem disease in patients with Pearson syndrome (Fig. 3). Historically, <20 % of patients were reported to have symptoms unrelated to bone marrow or gastrointestinal tract (Manea et al. 2009), but in our cohort, all Pearson syndrome patients had involvement of other systems. The renal tract was most commonly involved, with evidence of renal dysfunction in five Pearson syndrome cases, including Fanconi-type tubulopathy in two cases (G and H) and profound glomerular impairment in two (A and C). Renal involvement was not confined to those children with haematological problems, and overall, 17 of 20 (85 %) patients for whom results were available had abnormal glomerular and/or tubular dysfunction. The majority of patients had proximal tubulopathy, but other renal manifestations included glomerular compromise (A, K, L and W), nephrocalcinosis (diagnosed on autopsy in E and by ultrasound in W). End-stage renal failure occurred in one patient (K).

Our study demonstrates the relatively high frequency of renal disease in patients with mtDNA deletions. This is particularly interesting in light of previous observations of progressive renal failure in a murine model with mtDNA deletions (Inoue et al. 2000) and validates this model for preclinical trials of novel therapies for mtDNA deletion disorders. Furthermore, the utility of highly sensitive urinary markers of tubular damage, NAG and RBP (Herget-Rosenthal et al. 2004; Vaidya et al. 2008), allows presymptomatic detection of renal involvement and, consequently, early intervention. The presence of renal tubulopathy as detected by NAG/creatinine and RBP/creatinine ratios can also be a useful diagnostic clue and serve to increase clinical suspicion of an underlying mitochondrial disorder.

Ten of the 23 patients whose presentation was not secondary to anaemia fulfilled clinical diagnostic criteria for KSS. Patients U, X and AD were classified as having PEO and patients P, Q, Y, AA, AB, AE and AH as PEO+, since ptosis is recognised to precede ophthalmoplegia by several years (Jackson et al. 1995). In 15 of the 16 patients with ptosis, it was the only clinical symptom at initial presentation. Since SLSMDs can be detected noninvasively in children (on blood or urine analysis), we recommend that all children >1 year with undiagnosed new-onset ptosis should be screened for SLSMDs. It is to be noted, however, that ptosis in patient F was present at birth, and thus SLSMDs should be considered even in patients with apparent congenital ptosis, particularly if other systems subsequently become involved.

Cardiac involvement is well recognised in KSS and reported to affect almost 60 % of cases (Berenberg et al. 1977). Cardiac manifestations are less well known in Pearson syndrome (Rahman and Leonard 2000; Krauch et al. 2002; Akaike et al. 1997), but we observed cardiac involvement in three of 11 (27 %) children: two had rhythm disturbances: patient I, and patient B previously reported by Rahman et al. (Rahman and Leonard 2000); one (H) had RVH. Cardiac manifestations in other phenotypic subgroups were two KSS and three PEO+ patients (all had conduction defects), and three in the unclassified group (K and R had LVH; L had atrial fibrillation).

The spectrum of neurological presentations in childhood SLSMDs is constantly increasing (Morel et al. 2009; Lee et al. 2007). In the study reported here, the major documented neurological findings were generalised hypotonia and muscle weakness, with resultant delay in gross motor development in five patients. Neuromuscular symptoms were more common in patients without haematological impairment, possibly reflecting the high mortality rate in patients with Pearson syndrome, i.e. patients died before onset of neuromuscular symptoms. Early mortality of Pearson patients likely also explains the predominance of brain imaging abnormalities in patients without bone marrow manifestations. The most frequently observed brain MRI abnormalities were basal ganglia and white matter lesions. A notable exception was patient R, who appeared to show signs of impaired neuronal migration. To our knowledge, this is the first time an mtDNA deletion has been associated with imaging changes suggestive of a neuronal migration disorder, although other defects of mitochondrial oxidative phosphorylation (OXPHOS) function have been seen in patients with neuronal migration defects (van Straaten et al. 2005). As previously reported (McShane et al. 1991), we observed progression from Pearson syndrome to KSS in the two survivors who had long-term follow up.

Three patients, K, L and R, had no obvious external ophthalmoplegia or ptosis and, since they also did not have haematological compromise, fell outside any of the classical SLSMD phenotypes. They were diagnosed during investigation for other problems: faltering growth (K), Fanconi syndrome (L) and persistent neonatal lactic acidosis (R). Since patients with multisystem involvement are increasingly being investigated for mitochondrial disorders, it is likely that in the future a greater number of children will fall into this ill-defined group and new phenotypes of SLSMDs will emerge.

The range of clinical phenotypes was reflected in the varied need for symptomatic support. The need for careful endocrinology monitoring is evidenced by the requirement for hormone replacement in 12 of our cases: one required thyroxine, four cortisol replacement therapy, three vitamin D for hypoparathyroidism and three growth hormone. However, as might be expected, the most common endocrine abnormality was diabetes mellitus; five of the nine patients with abnormal glucose tolerance tests required insulin. Patients with impaired pancreatic exocrine function required pancreatic enzyme replacement. The other commonly compromised systems were cardiac (pacing was required in five patients) and renal: eight patients needed medical therapy (electrolyte replacement, in very high doses in some cases), one had renal replacement therapy while being considered for a transplant and one had lithotripsy to treat renal calculi. Finally, the importance of regular audiometry is emphasised by the finding of impaired hearing in ten patients and the need for cochlear implantation in one. This degree of multidisciplinary input, with contribution to management by specialist audiological physicians, cardiologists, endocrinologists, gastroenterologists, haematologists, nephrologists, neurologists, ophthalmologists and palliative care physicians, again emphasises the need for coordinated care of children with SLSMDs at a tertiary specialist centre.

Overall, our data provides further support for a clinical continuum of syndromes associated with SLSMDs manifesting in childhood. However, subdivision into Pearson syndrome and KSS may be useful for prognosis, as demonstrated by survival analysis of this cohort (Fig. 2). Five-year survival in patients manifesting with a Pearson phenotype was <50 % from time of initial presentation compared with 100 % for other phenotypic subgroups (*P* < 0.001). Although this mortality rate is high, it is considerably lower than a historical report of 76 % 5-year mortality (Rotig et al. 1995). Interestingly, survival at 4 years was 55 % in a more recent cohort (Manea et al. 2009), suggesting the possibility that earlier recognition of Pearson syndrome and intense management of known complications may be improving survival rates. Furthermore, all patients with Pearson syndrome who survived to 8 years were still alive at 18 years, which may be a useful prognostic feature when counselling parents and important information in planning transition to adult services.

Genotype–phenotype correlation for SLSMDs remains controversial. Traditionally, there was thought to be no relationship between the length of mtDNA deletion and clinical phenotype (Lopez-Gallardo et al. 2009; Rotig et al. 1995; Aure et al. 2007). However more recent work suggests that location (Lopez-Gallardo et al. 2009; Yamashita et al. 2008) and number of deleted tRNA molecules (Yamashita et al. 2008) may possibly influence phenotype, and a study of 87 patients (only five of whom presented younger than 20 years) indicated that mtDNA deletion size was correlated with both age at onset and progression rate. However, the impact of deletion size was mediated by the degree of heteroplasmy seen in muscle biopsy (Grady et al. 2014). It also suggested that deletion of the *MT-CYB* gene was associated with a more severe phenotype. In contrast, in our cohort, we found no correlation between patient age and deletion size or *MT-CYB* deletion. There was a significant, if minor, statistical correlation between degree of heteroplasmy and age at presentation. However, since quantitation was performed using visual inspection, this finding should be interpreted with caution.

## Conclusions

This retrospective cohort of patients with childhood-onset SLSMDs provides further evidence for a continuous clinical spectrum of disease associated with this genetic defect, including the occurrence of atypical presentations. Clinicians (including general paediatricians, neonatologists, ophthalmologists, renal physicians, endocrinologists, gastroenterologists, haematologists, child neurologists and paediatric metabolic specialists) should be aware of these heterogeneous presentations and maintain a high degree of suspicion for SLSMDs. Clinical features that should particularly raise suspicion of SLSMDs in children include sideroblastic anaemia, ptosis and multisystem disease with neurological, cardiac, renal and/or gastrointestinal manifestations. We find no correlation between deletion size and location and phenotype, but clinical categorisation into Pearson syndrome, KSS or PEO continues to be an important tool for prognostication. Longer survival of Pearson syndrome patients in this cohort compared with historical cohorts emphasises the importance of proactively monitoring for and aggressively managing known multisystem complications, including cardiac conduction defects, diabetes mellitus and renal impairment.

## Electronic supplementary material

Below is the link to the electronic supplementary material.

Table S1 (DOC 40 kb)

Table S2 (DOC 78 kb)

Table S3 (DOC 115 kb)

Table S4 (DOC 78 kb)
