# Supplementary material for: Paediatric single mitochondrial DNA deletion disorders: an overlapping spectrum of disease
Source: J Inherit Metab Dis. 2014 Oct 29;38(3):445–57. doi: 10.1007/s10545-014-9778-4 (PMC4432108; doi:10.1007/s10545-014-9778-4)
Supplement: Supplementary file 1 — (DOC 40 kb) [file 10545_2014_9778_MOESM1_ESM.doc]

**Table S1: Haematological features in patients with Pearson syndrome**

| **Patient** | **Onset of anaemia** | **Bone marrow** | **Neutropaenia** | **Onset of neutropaenia** | **Thrombocytopaenia** |
| --- | --- | --- | --- | --- | --- |
| A | Birth | Moderate dyserythopoesis, vacuolated myeloid precursors, ringed sideroblasts | Yes | Birth | No |
| B | Birth | Hypocellular, vacuolated precursors, ringed sideroblasts | Yes | Birth | Yes |
| C | Birth | Mild aplasia, vacuolated precursors, no sideroblasts | Yes | 4 months | Yes |
| D | Birth | Not available | Yes | Birth | Yes |
| E | Birth | Hypocellular, vacuolated precursors, ringed sideroblasts | Yes | Birth | Yes |
| F | Birth | Hypocellular, vacuolated precursors, no sideroblasts | No |  | Yes |
| G | Birth | Hypocellular, vacuolated precursors, ringed sideroblasts | Yes | Birth | Yes |
| H | 2 months | Hypocellular, vacuolated precursors, ringed sideroblasts | Yes | 1 years | Yes |
| I | 5 months | Hypocellular, vacuolated precursors, ringed sideroblasts | Yes | 2 years | Yes |
| J | 16 months | Hypocellular, vacuolated precursors, no sideroblasts | Yes | 18 months | No |
| AG | 4 months | Hypocellular, vacuolated precursors, ringed sideroblasts | Yes | 5 months | No |
