# Supplementary material for: Paediatric single mitochondrial DNA deletion disorders: an overlapping spectrum of disease
Source: J Inherit Metab Dis. 2014 Oct 29;38(3):445–57. doi: 10.1007/s10545-014-9778-4 (PMC4432108; doi:10.1007/s10545-014-9778-4)
Supplement: Supplementary file 2 — (DOC 78 kb) [file 10545_2014_9778_MOESM2_ESM.doc]

**Table S2: Gastrointestinal and endocrine features**

| **Patient** | **Weight centile** | **Height centile** | **Pancreatic insufficiency** | **Chronic diarrhoea** | **Diabetes mellitus** | **Other endocrine problems** |
| --- | --- | --- | --- | --- | --- | --- |
| A | 3^rd^🡪<<3^rd^ | <3^rd^🡪<<3^rd^ | Yes by 4y 2m | No | Yes at 4y | Hypothyroid at 5y |
| B | 75^th^🡪 <2^nd^ | 25^th^🡪 <3^rd^ | Yes by 5y | No | No |  |
| C | 10^th^🡪<3^rd^ | 25^th^🡪<3^rd^ |  | No |  | Normal TFTs |
| D | 3rd🡪<3^rd^ (22m) | <<3^rd^ (22m) |  | No |  | Normal TFTs |
| E | <3^rd^🡪<0.4^th^ | <0.4^th^ | No but hypoplastic pancreas on autopsy | No | Yes |  |
| F | - | - | - | No | - | Normal TFTs |
| G | 10^th^🡪<3^rd^ (8m) |  | Yes by 2y | Yes | Yes | Normal TFTs, adrenal function |
| H | 3^rd^🡪<<0.4^th^ | <0.4^th^ | No | Yes | - | Normal TFTs |
| I |  |  | Yes by 2y | Yes | - |  |
| J | 🡪50^th^ | 9^th^ | No | Yes | No | Normal TFTs, hypoparathyoidism at 6y |
| K | 50^th^🡪9th | 0.4^th^ | Yes | Yes | Yes at 15m | Normal TFTs |
| L | 🡪<0.4^th^ | <0.4^th^ | - | No | Yes at 13m | - |
| M | 🡪2^nd^ | 2^nd^ | - | No | No | Growth hormone deficiency: IGF1 = 53 ng/ml (reference 74-388), IGFBP3 = 1.87 mg/L (1.8-7.1), Glucagon stimulation test showed peak growth hormone 2.5 mcg/L |
| N | 🡪2^nd^ | 0.4^th^🡪2^nd^ | No | No | Yes at 10y | Normal TFTs |
| O | <3^rd^ (18 m) | 10^th^ | No | No | No | - |
| P | <3^rd^ 🡪<2^nd^ | <2^nd^ | - |  | No | Adrenal insufficiency, normal TFTs |
| Q | <3^rd^ 🡪below 2nd | < 2^nd^ | - | No | - | Adrenal insufficiency, normal TFTS |
| R | <2^nd^ | <2^nd^ | Required neocate to thrive but elastase not measured | Yes | No | Normal TFTs and cortisol |
| S | 25^th^ | 25^th^-50^th^ | - | No | - | Normal TFTs |
| T | 25^th^ | 25^th^ | - | No | No | Normal TFTs |
| U | 25^th^ | 25^th^ | No | No | No | Normal TFTs |
| V | <3^rd^ | <3^rd^ | No | No | Yes 14y | Hypoparathyroid from 12y  Normal TFTs |
| W | 9^th^🡪3rd | 25^th^🡪 3^rd^ | No | No | Yes 15y | Adrenal insufficiency 7 y, hypoparathyroidism 7 y  Growth hormone insufficiency  Normal TFTs |
| X | 50^th^ | 50^th^ | - | No | - | Normal TFTs |
| Y | 50^th^ | 50^th^ | - | No | - | - |
| Z | <3^rd^🡪<3rd | <3^rd^🡪<3rd | - | No | No | Normal TFTs, GH, adrenal |
| AA | 50th | 75th | - | No | No | Normal TFTs |
| AB | 50^th^🡪<3rd | 50^th^🡪<3^rd^ (NB scoliosis) | - | No | No | - |
| AC | 75^th^🡪75^th^ | 50^th^🡪25th | - | No | No | Normal TFTs |
| AD |  |  | - | No | No | Normal TFTs |
| AE | 50^th^🡪25^th^ | 50🡪3^rd^ | - | No | No | Normal TFTs |
| AF | 3^rd^🡪<<0.4^th^ | <0.4 | No | No | Yes 8.5y | Normal TFTs, Growth Hormone deficiency |
| AG | 75🡪 25th | 75^th^🡪 25 | no | no | no | Normal TFTs |
| AH | - | - | - | - | - | Normal TFTs |

Key: IGF1 = insulin-like growth factor 1; IGFBP3 = insulin-like growth factor-binding protein 3; m = months; TFTs = thyroid function tests; y = years; - denotes results unavailable
