# Supplementary material for: Paediatric single mitochondrial DNA deletion disorders: an overlapping spectrum of disease
Source: J Inherit Metab Dis. 2014 Oct 29;38(3):445–57. doi: 10.1007/s10545-014-9778-4 (PMC4432108; doi:10.1007/s10545-014-9778-4)
Supplement: Supplementary file 3 — (DOC 115 kb) [file 10545_2014_9778_MOESM3_ESM.doc]

**Table S3: Renal features**

| **Patient** | **Glomerular function (formal EDTA-Cr evaluation, normal >90ml/min/1.73m^2^)** | **Tubular function** | | | **Other** | **Renal biopsy** |
| --- | --- | --- | --- | --- | --- | --- |
|  |  | **NAG/Cr**  **(normal range 2-12 mmol/mmol creatinine)** | **RBP/Cr**  **(normal range 3.9-32 mmol/mmol creatinine)** | **Total Reabsorption of Phosphate (normal >80%)** |  |  |
| A | 41 (4y 2m) | 42 (18m) 🡪36 (36 m) | 81 (18m) 🡪3500 (36m) | - | - | Normal |
| B | - | 48 (3y) 🡪103 (6y) | 2662 (6y) | Normal | - | Normal |
| C | 98 (4y)🡪 51 (6y) 🡪25 (7y) | - | - | - | - | Interstitial fibrosis/foci of calcinosis at 4y |
| E | - | - | - | - | - | Widespread cystic dilatation of cortical tubules |
| F | 115 (5y) | - | 13, 23 at 5y | Normal | - | - |
| G | - | - | - | - | Polydipsia, polyuria, aminoaciduria + glycosuria | - |
| H | - | - | - | - | Generalized aminoaciduria, polydipsia + polyuria | - |
| I | - | 45 (4y) 🡪75 (5y) | 17 (4y) 🡪54 (5y) | Normal (4y + 6y) | - | - |
| J | - | 42 | 1844 (6y) | - | - | - |
| K | 29 (6 y), 27 (14y) | 103 (20m) 🡪109 (15y) | 3659 (15y) | Normal | - | Normal |
| L | 43 (15m) | 80 (10y)🡪 90 (11 y) | 5476 (14y)🡪3717 (15y) | Normal (10y) 🡪63% (11y) | - | Normal |
| M | - | 125 | 5265(10y) | - | - | - |
| N | - | 42 | 14 | Normal | - | - |
| O | - | 117 | 101 | Normal (16y) | - | - |
| P | - | 63🡪53 | 104🡪257 | - | - | - |
| Q | - | 56 | 66 | Normal | - | - |
| R | - | 97 | 34 | Normal | - | - |
| S | 105 | - | - | Normal | - | - |
| V | - | 5 | 23 | Normal | - | - |
| W | 70 at 14 | 341 at 15 | 3401 (15 y) | 73% (15 y) | - | Nephrocalcinosis also seen on U/S + renal calculi |
| X | >90 | - | - | - | - | - |
| Z | - | 20 | 5 | Normal | - | - |
| AG | - | 65 | 55 | - | - | - |

Key: m = months; NAG/Cr = N- acetyl-glucosaminidase; RBP/Cr = Retinol binding protein/creatinine; y = years; patients not included had no investigations performed; - denotes results unavailable

**Table S4**: Biochemical features

| **Patient** | **Plasma lactate (mmol/L)** | **CSF lactate (mmol/L)** | **CSF protein (mmol/L) +**  **5-MTHF (nmol/L)** | **Hypoglycaemia** | **Plasma amino acids** | **Blood spot acylcarnitines** | **Urine organic acids** |
| --- | --- | --- | --- | --- | --- | --- | --- |
| A | 4.9 | - | - | - | - | Normal | - |
| B | 2.9 🡪 7.3 (5y) | - | - | No | Alanine =840 | - | 3-hydroxybutyrate, lactate, fumarate, malate and citrate |
| C | 3 (20m)🡪1.8 (4y 2m) | - | 1.72 | - | Normal alanine | - | Lactate, 3-hydroxybutyrate |
| D | 4.2 (3m) 🡪 3.4 at 20m, 8 (22m) | - | - | - | - | - | - |
| E | 4.33 | - |  | No |  | - | - |
| F | 2.4 🡪4.7 (5y) | 5.5 | - | No | - | - | - |
| G | 6.3 to 7.3 (4m)🡪11.2 (13m) | - | - | Yes | - | - | Normal at 13m |
| H | 8 at 1y🡪 13.7 to 18.9 (18m) | - | - | No | Normal alanine | - | 3-hydroxybutyrate, 4-hydroxyphenyllactate |
| I | 2.5🡪2.9 | - | - | Yes | Alanine = 824 | Normal | 3-hydroxybutrate, 3-methylglutaconate, 3-methylglutarate |
| J | 1.86 (6.5y) | 2.3 | 1.99  5MTHF=10 (72-305) | Yes | Raised threonine  Normal alanine | Raised 4-hydroxybutryl carnitine | Moderately raised lactate, mildly raised pyruvate |
| K | 4.2-6.1, 2.8 ( 5y), 3 (16y) | 4.1 | 1.66  5MTHF= 9(rr 72-305) | No | Alanine =897 | Normal | - |
| L | 1.72 (14m) | - |  | No | - | Normal | Lactate, 2-hydroyxybutyrate , 3-hydroxybutyrate |
| M | 1.7 (6y) | 3.0 | 1.7 | Yes | Alanine= 623, proline= 323 | Raised 4-hydroxybutrylcarnitine | 3-hydroxybutyrate |
| N | 3.5(5y) | 3.7 | 2.52  5MTHF=7 ( rr 72-305) | No | Alanine= 732 | Normal | 3-hydroxybutyrate |
| O | 3.7 (6y) | 4.9 | - | No | Alanine= 889 | Normal | Lactate, 3-hydroxybutyrate |
| P | 2.1 to 2.9 (8y) | - | - | - | Alanine= 562 | - | Normal at 8y |
| Q | 2.1(8y) | 3.08 | 2.04 | No | Alanine =572 | Normal | - |
| R | 5.6-6.5 (birth) | 3.4 | 1.2 | Yes | Alanine =800 | Normal | Lactate, malate, fumurate, oxoglutate, 3 hydroxybuturate |
| S | 4.3 (17) | - | - | No | Alanine= 850 | - | - |
| T | <2 on repeat | Mild increase on MRI spectroscopy | - | No | Normal | Normal | Normal |
| U | <2 |  | - | - | - | - | - |
| V | 3.5 | - | - | Yes | Alanine =546 | Normal | - |
| W | <2 | - | 2.2 | No | Alanine =492 | Normal | 3-hydroxybutyrate with mildly raised 2-hydroxybutyrate, moderately raised lactate |
| X | - | - | - | No | - | - | - |
| Y |  | - |  | No | - | - | - |
| Z | 2.3🡪 3.6 | 2.4 | 0.85  5MTHF=54 (72-172) | No | Alanine=608 | Normal | Pyruvate, lactate |
| AA | 1.3 | - | - | No | - | - | - |
| AB | 1.8 | - | - | - | - | - | - |
| AC |  | - | - | - | - | - | - |
| AD | 1.3 | - | - | - | - | - | - |
| AE |  | - | - | - | - | - | - |
| AF | 1.8🡪 6.8 | 5.1 | 1.81 | - | Normal | Normal | Normal |
| AG | 2.3-> 2.9 | - | - | - | Alanine = 596 | Normal | Increased lactate |
| AH | 2.3 | - | - | - | Alanine = 510 | Normal | - |

Key: 5MTHF = 5-Methyltetrahydrofolate; m = months; y = years; - denotes results unavailable
