# Supplementary material for: Paediatric single mitochondrial DNA deletion disorders: an overlapping spectrum of disease
Source: J Inherit Metab Dis. 2014 Oct 29;38(3):445–57. doi: 10.1007/s10545-014-9778-4 (PMC4432108; doi:10.1007/s10545-014-9778-4)
Supplement: Supplementary file 4 — (DOC 78 kb) [file 10545_2014_9778_MOESM4_ESM.doc]

**Table S4**: Biochemical features

| **Patient** | **Plasma lactate (mmol/L)** | **CSF lactate (mmol/L)** | **CSF protein (mmol/L) +**  **5-MTHF (nmol/L)** | **Hypoglycaemia** | **Plasma amino acids** | **Blood spot acylcarnitines** | **Urine organic acids** |
| --- | --- | --- | --- | --- | --- | --- | --- |
| A | 4.9 | - | - | - | - | Normal | - |
| B | 2.9 🡪 7.3 (5y) | - | - | No | Alanine =840 | - | 3-hydroxybutyrate, lactate, fumarate, malate and citrate |
| C | 3 (20m)🡪1.8 (4y 2m) | - | 1.72 | - | Normal alanine | - | Lactate, 3-hydroxybutyrate |
| D | 4.2 (3m) 🡪 3.4 at 20m, 8 (22m) | - | - | - | - | - | - |
| E | 4.33 | - |  | No |  | - | - |
| F | 2.4 🡪4.7 (5y) | 5.5 | - | No | - | - | - |
| G | 6.3 to 7.3 (4m)🡪11.2 (13m) | - | - | Yes | - | - | Normal at 13m |
| H | 8 at 1y🡪 13.7 to 18.9 (18m) | - | - | No | Normal alanine | - | 3-hydroxybutyrate, 4-hydroxyphenyllactate |
| I | 2.5🡪2.9 | - | - | Yes | Alanine = 824 | Normal | 3-hydroxybutrate, 3-methylglutaconate, 3-methylglutarate |
| J | 1.86 (6.5y) | 2.3 | 1.99  5MTHF=10 (72-305) | Yes | Raised threonine  Normal alanine | Raised 4-hydroxybutryl carnitine | Moderately raised lactate, mildly raised pyruvate |
| K | 4.2-6.1, 2.8 ( 5y), 3 (16y) | 4.1 | 1.66  5MTHF= 9(rr 72-305) | No | Alanine =897 | Normal | - |
| L | 1.72 (14m) | - |  | No | - | Normal | Lactate, 2-hydroyxybutyrate , 3-hydroxybutyrate |
| M | 1.7 (6y) | 3.0 | 1.7 | Yes | Alanine= 623, proline= 323 | Raised 4-hydroxybutrylcarnitine | 3-hydroxybutyrate |
| N | 3.5(5y) | 3.7 | 2.52  5MTHF=7 ( rr 72-305) | No | Alanine= 732 | Normal | 3-hydroxybutyrate |
| O | 3.7 (6y) | 4.9 | - | No | Alanine= 889 | Normal | Lactate, 3-hydroxybutyrate |
| P | 2.1 to 2.9 (8y) | - | - | - | Alanine= 562 | - | Normal at 8y |
| Q | 2.1(8y) | 3.08 | 2.04 | No | Alanine =572 | Normal | - |
| R | 5.6-6.5 (birth) | 3.4 | 1.2 | Yes | Alanine =800 | Normal | Lactate, malate, fumurate, oxoglutate, 3 hydroxybuturate |
| S | 4.3 (17) | - | - | No | Alanine= 850 | - | - |
| T | <2 on repeat | Mild increase on MRI spectroscopy | - | No | Normal | Normal | Normal |
| U | <2 |  | - | - | - | - | - |
| V | 3.5 | - | - | Yes | Alanine =546 | Normal | - |
| W | <2 | - | 2.2 | No | Alanine =492 | Normal | 3-hydroxybutyrate with mildly raised 2-hydroxybutyrate, moderately raised lactate |
| X | - | - | - | No | - | - | - |
| Y |  | - |  | No | - | - | - |
| Z | 2.3🡪 3.6 | 2.4 | 0.85  5MTHF=54 (72-172) | No | Alanine=608 | Normal | Pyruvate, lactate |
| AA | 1.3 | - | - | No | - | - | - |
| AB | 1.8 | - | - | - | - | - | - |
| AC |  | - | - | - | - | - | - |
| AD | 1.3 | - | - | - | - | - | - |
| AE |  | - | - | - | - | - | - |
| AF | 1.8🡪 6.8 | 5.1 | 1.81 | - | Normal | Normal | Normal |
| AG | 2.3-> 2.9 | - | - | - | Alanine = 596 | Normal | Increased lactate |
| AH | 2.3 | - | - | - | Alanine = 510 | Normal | - |

Key: 5MTHF = 5-Methyltetrahydrofolate; m = months; y = years; - denotes results unavailable
